# Low-Cost Interrogation Technique for Dynamic Measurements with FBG-Based Devices

**DOI:** 10.3390/s17102414

**Published:** 2017-10-23

**Authors:** Camilo A. R. Díaz, Cátia Leitão, Carlos A. Marques, M. Fátima Domingues, Nélia Alberto, Maria José Pontes, Anselmo Frizera, Moisés R. N. Ribeiro, Paulo S. B. André, Paulo F. C. Antunes

**Affiliations:** 1 Telecommunications Laboratory LABTEL, Electrical Engineering Department, Federal University of Espírito Santo, 29075-910 Espírito Santo, Brazil; mjpontes@ele.ufes.br (M.J.P.); frizera@ieee.org (A.F.); moises@ele.ufes.br (M.R.N.R.); 2 Department of Physics & I3N, University of Aveiro, Campus Universitário de Santiago, 3810-193 Aveiro, Portugal; catia.leitao@ua.pt (C.L.); carlos.marques@ua.pt (C.A.M.); fatima.domingues@ua.pt (M.F.D.); pantunes@ua.pt (P.F.C.A.); 3 Instituto de Telecomunicações, Campus Universitário de Santiago, 3810-193 Aveiro, Portugal; nelia@ua.pt; 4 Centre for Mechanical Technology and Automation, Department of Mechanical Engineering, University of Aveiro, Campus Universitário de Santiago, 3810-193 Aveiro, Portugal; 5 Department of Electrical and Computer Engineering and Instituto de Telecomunicações, Instituto Superior Técnico, University of Lisbon, 1049-001 Lisbon, Portugal; paulo.andre@lx.it.pt

**Keywords:** interrogation technique, fiber Bragg grating (FBG), in-line Fabry–Perot interferometer (FPI), optical accelerometer, arterial central pulse waveform, cardiovascular monitoring

## Abstract

Fiber Bragg gratings are widely used optical fiber sensors for measuring temperature and/or mechanical strain. Nevertheless, the high cost of the interrogation systems is the most important drawback for their large commercial application. In this work, an in-line Fabry–Perot interferometer based edge filter is explored in the interrogation of fiber Bragg grating dynamic measurements up to 5 kHz. Two devices an accelerometer and an arterial pulse wave probe were interrogated with the developed approach and the results were compared with a commercial interrogation monitor. The data obtained with the edge filter are in agreement with the commercial device, with a maximum RMSE of 0.05 being able to meet the requirements of the measurements. Resolutions of 3.6 pm and 2.4 pm were obtained, using the optical accelerometer and the arterial pulse wave probe, respectively.

## 1. Introduction

The popularization and fast growth of optical fiber sensing technology has stimulated much research in different areas, such as industrial, medical, aerospace, civil, and so on, where measurements of diverse physical and chemical parameters, such as acceleration, level, temperature, strain, pressure, deformation, refractive index, among others, are required. Optical fiber sensors (OFSs) have been developed and broadly used for these measurements [1,2,3,4,5,6]. This technology has important features such as intrinsic safety, resistance to chemical corrosion, immunity to electromagnetic interference, electric isolation, small size, lightweight sensing heads, high resolution, easy multiplexing, and capability for extremely remote monitoring without the need of electrical power at the measuring point [7,8]. Among the OFSs, fiber Bragg gratings (FBGs) are by far the most used for measuring temperature and/or mechanical strain [9,10].

FBGs are nanometer periodical refractive index changes engraved in an optical fiber core. When a broadband light spectrum is injected in the fiber, this optical signal will interact with the FBG, where the wavelengths that fulfill its resonation condition are reflected, while the others are transmitted. The reflected spectrum is centered at the Bragg wavelength whereas in the transmitted signal a suppression can be seen at the same wavelength [11]. The same operation principle can be observed in novel and more complex structures such as planar Bragg grating sensors [12,13]. The FBGs provide highly accurate measurements, since wavelength is an unalterable property of the signal along the optical fibers. Nonetheless, the interrogation systems are the most important drawback for their large commercial application, due to their high cost. Therefore, the development of new, and lower cost interrogation alternatives is essential [14].

The FBG sensors spectrum is usually monitored either by an optical spectrum analyzer (OSA) or a commercial FBG interrogation system. For real-time application in industry, the OSA is not suitable due to trade-offs between resolution and sweep frequency, along with OSA cost, volume and weight [15]. Commercial OFS interrogators (designed based on a scanning laser or scanning filters) are able to probe the FBG spectrum with higher resolution and scanning frequency. However, they become extremely expensive whenever hundreds of scans per second are required [16]. Several solutions for dynamic monitoring have been proposed. A fast and lower cost FBG interrogation technique is frequency-to-amplitude conversion, usually referred to as edge filtering, where the FBG’s spectral variations are straightforwardly translated into optical power variations [17,18]. This technique is based on the convolution between both the FBG sensor and the edge filter spectra. The filtering process is usually performed by a second FBG, the edge filter itself, unbalanced interferometers, Fabry–Perot filters, among others [19,20]. Standard FBG used as an edge filter shows high sensitivity but very limited dynamic range [20,21,22]. Tilted FBG required special treatment in order to obtain high visibility [23]. Long period gratings exhibit a large dynamic range which would limit measurement accuracy and the number of sensors which can be multiplexed [24].

This work presents a low-cost FBG interrogator system for dynamic measurements which is constructed with a simple, compact, stable and inexpensive in-fiber solution based on catastrophic fuse effect micro-cavity Fabry–Perot interferometers (FPIs) used as an edge filter [2,25,26]. The main component of the proposed system interrogation is constructed by using a commercial splice machine, standard single mode fiber (SMF), and recycled fiber destroyed by the catastrophic fuse effect. This paper is focused on two different dynamic test applications based on FBGs, an accelerometer and a non-invasive carotid pulse waveform probe. The studied sensors have been broadly researched [27,28,29,30], and the monitoring data is compared with the commercial interrogation technique used by the authors in the referred publications.

## 2. Materials and Methods

### 2.1. Proposed Interrogator Operation Principle

**Figure 1:**
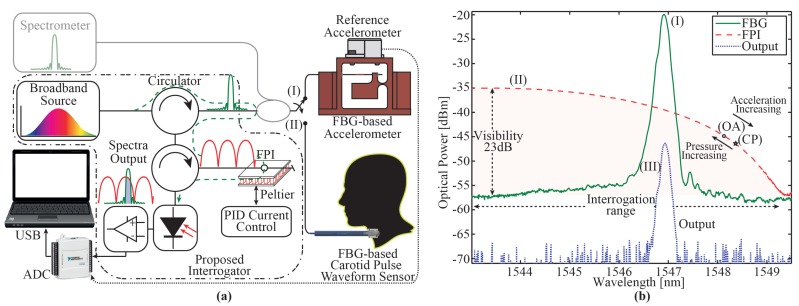
(**a**) Setup of the proposed interrogator; (**b**) Spectra of the proposed interrogation system, acquired by an optical spectrum analyzer (OSA). (I), (II) and (III) represent the spectra of fiber Bragg grating (FBG), Fabry–Perot interferometer (FPI), and output, respectively. (OA) and (CP) are the central wavelength of the optical accelerometer and carotid pulse waveform sensor, respectively.

The low-cost FBG interrogator system depicted in the dashed–dotted area of Figure 1a is based on three main optical devices: the light source (ALS-CL-17-B-FA, Amonics, Hong Kong, China), two circulators (CIR-3-SCL-1-FA, OeMarket, Galston, Australia), and one photodetector (GAP 10 FC 0622, GPD Optoelectronics Corp., Salem, NH, USA). The amplified spontaneous emission (ASE) broadband light source from 1520 nm to 1580 nm, with -20 dBm of optical power is launched to the first circulator, where a splitter 99/1 (TWBCB101PS210, Hammondmfg) is added to allow monitoring of the backscattered spectrum of the FBG-based sensor by both the spectrometer (I-MON 512E-USB, Ibsen, Farum, Denmark) with a sample rate of 945 Hz (1% optical power), and the proposed low-cost interrogator (99% of optical power). The backscattered spectrum of the FBG sensor is coupled to the in-line FPI by using a second optical circulator. Thus, the resulting optical power (output), the product of the convolution between both spectra of the FBG and FPI, is detected by the photodetector which is, in turn, amplified and posteriorly acquired by an analog-to-digital converter (ADC) system (USB6008, National Instruments, Ennetbaden, Switzerland), which provides a maximum data acquisition rate of 10,000 samples per second (sps), with an input dynamic range of ±10 V and a 12-bit resolution. The acquisition rate could be improved by changing this module for modern electronic devices, e.g., microcontrollers, allowing to achieve higher acquisition rates up to around 500 ksps with 16-bits ADC conversion and several channels. The in-line FPI micro-cavity is housed in a copper structure to reduce external mechanical deformation and vibration. This special micro-cavity conveniently shows low temperature sensitivity compared to FBGs [25]. However, a temperature control, with a Peltier plate (in a simple configuration just to keep the temperature constant), is used to improve the interrogation accuracy of this FPI-based method. Therefore, only the FBG will be exposed to external perturbations, which induces fiber Bragg wavelength shifts, translated into output optical power variation by the FPI. As depicted in Figure 1a, both FBG-based sensors use the same setup, however, in the case of the optical accelerometer (I), two ACD channels are measured: one channel for the optical accelerometer and the other for an electronic accelerometer, which is used as a second reference (dotted line). Considering the Nyquist theorem, the sampling rate must be at least two times the maximum frequency to be detected. It means that the highest measurable frequency is 5 kHz. In the case of the optical accelerometer, two channels have been used, limiting the measurable frequency to 2.5 kHz. To perform both experiments, the measure amplitude range was selected as 5 VDC and the sample rate was 5 ksps. 

Both monitored FBGs were recorded in photosensitive SMF (GF1B, ThorLabs, Newton, NJ, USA) by the phase mask technique with a KrF UV Excimer laser emitting at 248 nm (BraggStar Industrial, Coherent, Santa Clara, CA, USA). The FBGs were inscribed with 5 mJ energy pulses and a repetition frequency of 500 Hz. FBGs of 10 mm in length were selected to obtain more than 90% of reflectivity [31]. The Bragg wavelength selected for both sensors was around 1547.0 nm. The FPI micro-cavity was fabricated with a similar process as presented in [25]. A commercial fusion splice machine (50S, Fujikura, Tokyo, Japan), a standard SMF (SMF-125/9, Corning, New York, NY, USA) and a fiber (SMG-652, Corning), which was recycled after destroyed by the catastrophic fiber fuse effect (produced with a Raman fiber laser IPG, Model RLR-10- 1480 at 3 W of optical power) [25,32,33], were used to create the micro-cavity. An approximation of the physical dimensions of the micro-cavity, measured from an image taken by electronic microscopy, with amplification of x 50, is 80.1 μm in width, and 122.8 μm in height. The resulting spectrum depicted high visibility around 23 dB and a free spectrum range around 13 nm.

Figure 1b shows the optical spectra (OSA with 50 pm resolution, AQ6375, Yokogawa, Tokyo, Japan) of the FBG, in-line FPI, and the convolution between them (output), used in this low-cost FBG interrogation approach. First, the input ASE through the first circulator and the FBG backscattered optical spectrum can be observed. The Bragg wavelength is centered at around 1547.0 nm with more than 30 dB of contrast. The central Bragg wavelength of each sensor is shifted to 1548.2 nm and 1548.3 nm, for the optical accelerometer (OA) and the carotid pulse waveform sensor (CP), due to the sensors pretension before being glued in the supporting structures. Second, the ASE signal goes to the second circulator, and to the FPI operating in reflection mode (II). As observed in the highlighted region, there are two important characteristics enabling the micro-cavity to be used as an edge filter interrogator: the high contrast (23 dB) and the interrogation range of 6 nm (from 1543.4 nm to 1549.4 nm). Finally, all devices are combined and the output spectrum is obtained, which is the result of both FBG and FPI spectra filtering (III). Due to the periodicity of the FPI’s optical spectrum, the interrogation range is limited to 50% of the FPI’s free spectrum range (FSR). Considering a standard FBG sensor, which has a strain sensitivity around 1.2 pm/με for 1550 nm, our edge filtering technique allows the FBG sensor to be stressed to nearly 5000 με. It is higher than the maximum value of strain supported by a standard FBG sensor, and consequently high enough for the applications here presented, where the maximum strain value is in the range of hundreds of pm. Due to the shape of the FPI spectrum being non-linear, depending on the position of the central wavelength of the FBG in the interrogation range, the sensitivity is affected. If the OA and CP points are located close to the peak of the spectrum, the sensitivity will be reduced considerably; otherwise, if these points are positioned at the valley, sensitivity will be increased but the optical power will be reduced, requiring more sensitivity of the photodetector. In addition, the behavior of the sensors is opposite; this means that when the optical accelerometer is exposed to an external acceleration, redshift wavelength will be induced. On the other hand, when the central arterial pulse wave probe head is exposed to external pressure, blueshift wavelength is produced. Therefore, it is convenient to leave a dead zone between the Bragg wavelength and the peak and valley of the FPI micro-cavity spectra.

### 2.2. FBG-Based Sensors Used to Test the Interrogation System

**Figure 2:**
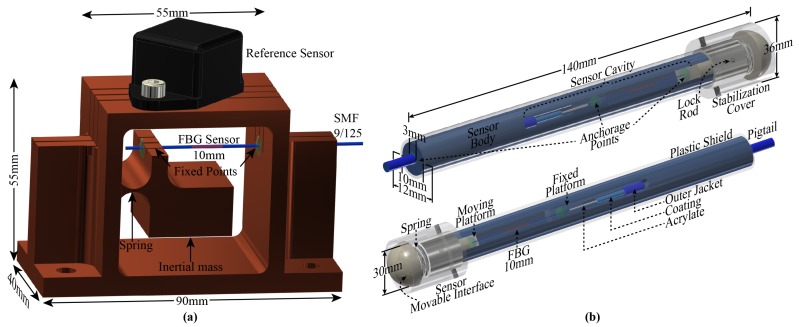
Schematics of the FBG-based sensors: (**a**) Optical accelerometer; (**b**) Central arterial pulse wave probe sensor.

Figure 2a depicts the FBG-based accelerometer, where the Bragg grating is embedded into a cantilever-based structure which was manufactured form a bulk brass piece. In this test, an electronic accelerometer was fixed over the top side of the structure and used as a reference sensor, in addition to the commercial spectrometer. The operation principle of the accelerometer is based on the vertical movement of the concentrated inertial mass which imposes a contraction and an expansion in the FBG sensor when it is exposed to external accelerations [34]. As observed in Figure 2a, both the inertial mass and the FBG are housed in a central cavity of the support structure. 

Figure 2b depicts the FBG-based carotid arterial pressure wave profile sensor head. The operation principle of the proposed probe is based on the longitudinal movement of the sensor movable interface (SMI) which imposes contraction and expansion of the FBG sensor when it is exposed to external pressure [28,29,30]. The sensor body is an aluminum pipe which has two holes (with different diameters and depths) in its extremities and one longitudinal cavity which allows both positioning and anchoring the fiber with the FBG inside the probe body. As the moving platform is pushed in or out of the sensor body, it will provoke a compression/stretching of the FBG, resulting in a Bragg wavelength shift that will induce optical power variations.

## 3. Results

### 3.1. Optical Accelerometer

In order to characterize the optical accelerometer, two experiments were conducted at room temperature (∼22 °C). First, the frequency response of the system was determined. The inertial mass was displaced from the equilibrium position by applying force over the mass in the vertical direction. Subsequently, the mass was left in free vibration response in the absence of external loading. Second, since an acceleration-controlled measurement system is not available, the optical accelerometer was fixed to a solid aluminum optical breadboard (SAOB) that was exposed to an almost constant oscillation. In both experiments, three signals were monitored simultaneously.

**Figure 3:**
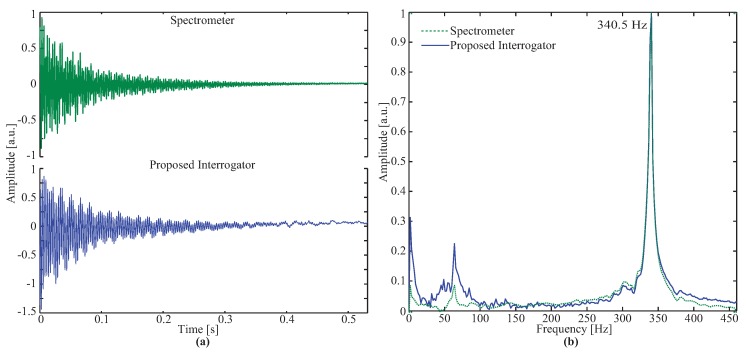
Natural response of the optical accelerometer: (**a**) Time domain response; (**b**) Frequency domain response.

Figure 3a depicts the normalized temporal natural response of the optical accelerometer measured with the spectrometer (top side) and the proposed interrogator (bottom side). It can be observed that the damping time after the mass is left in free vibration in the absence of external loading is approximately 0.4 s. Due to the proposed interrogation system having a higher sampling rate (more than five times) than the commercial spectrometer, a lower frequency component is more evident in the proposed interrogator response. 

The reference signal from the electronic accelerometer is not presented, because the experiment was conducted in a static condition by directly displacing the inertial mass instead of the whole accelerometer structure, therefore, the electronic sensor was not submitted to any acceleration. The fast Fourier transform (FFT) of the temporal response is depicted in Figure 3b, as observed by the natural response of the optical accelerometer which is around 340.5 Hz, agreeing with [27], with a relative error of 1%. In the responses of both the spectrometer and developed interrogator, a second harmonic appears around 63.1 Hz. This component is due to the fact that the perturbation over the mass was applied in a corner instead of in the center of the mass, thus also exciting a transversal vibration component.

**Figure 4:**
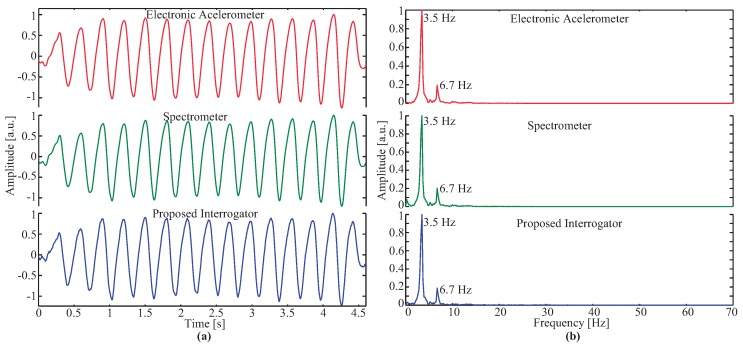
Response of quasi-periodic oscillation: (**a**) Temporal result; (**b**) fast Fourier transform (FFT) of the temporal response.

The results from the second experiment are presented in Figure 4a, where the three normalized temporal signals obtained during platform oscillation are compared. As observed, the proposed low-cost interrogator system measures the oscillation accordingly with both the references, electronic accelerometer and the spectrometer. The root-mean-square error (RMSE) between the electronic accelerometer signal and the optical signals from the spectrometer and the proposed interrogator were 0.048 and 0.047, respectively, and 0.039 when compared to the spectrometer with the proposed technique. The voltage shift in the developed interrogator was found to be around ∼20 mVpp and the wavelength shift obtained by the commercial spectrometer was approximately ∼60 pm. The relation between both was determined as ∼0.3 mV/pm which is higher than the result obtained in [34]. The resolution of the system has been determined as ∼3.6 pm. The frequency response for the second experiment was analyzed by performing a FFT and it is depicted in Figure 4b. The main frequency component determined was ∼3.5 Hz. As observed in the three signals, a second frequency component appears at ∼6.7 Hz. The presence of this component can be justified by the asynchronous movement in the extremities of the SAOB to alternate the movement in the opposite direction, i.e., from up to down and vice-versa. 

### 3.2. Carotid Pulse Waveform FBG-Based Device

**Figure 5:**
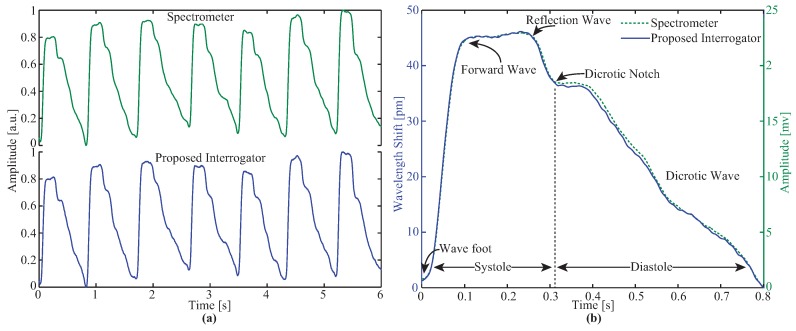
Signals acquired by the spectrometer and the proposed interrogator: (**a**) Sequence of seven pulses; (**b**) Comparison of a single pulse.

In order to compare the responses of both interrogators, the pulse wave was accessed in a healthy 36-year-old human female. First, the carotid location with major pulsation amplitude was searched, suggesting the place where the sensor must be placed to access the arterial pressure waveform. The waveforms were acquired with both the commercial spectrometer and the proposed interrogator for 50 s. The experiment was performed at room temperature ∼22 °C. The normalized and filtered carotid pulse waveforms of both the spectrometer and the proposed low-cost interrogator system are depicted in Figure 5a. A first-order bandpass filter from 0.3 Hz to 20.0 Hz was used (typical filtering used in these data acquisitions) in order to minimize both the optical noise and the drifting of the measured signal baseline related with temperature and pressure (from the user during the measurement) effects. From the analyzed signal, seven consecutive pulses are presented, where only negligible deviations between the signals are detected. The RMSE between the two signals was determined to be 0.010, showing that the proposed interrogation technique is reliable for this application. Figure 5b shows a single pulse without normalization. The wavelength shift is around 46 pm and the peak-to-peak voltage variation is approximately 23 mV. The relation between the voltage and the wavelength is 0.5 mV/pm. With the used ADC module (12-bits), it is possible to achieve a resolution of 2.4 pm which could be improved if the resolution of the ADC is increased. If a 16-bit ADC were used, a 0.15 pm resolution could be reached. As observed in Figure 5b, the proposed interrogator response matches the reference signal (spectrometer), allowing the specific points on the carotid pulse to be detected: the wave foot, the forward, reflection and dicrotic waves, and the dicrotic notch. Furthermore, the lower cost interrogation technique is suitable for all the sensors presented in the state-of-the-art [28,29,30,35].

### 3.3. Temperature Cross Sensitivity Discussion

FBG sensors usually present a temperature sensitivity around 10 pm/°C. Due to the edge filter’s spectrum being non-linear, depending on the position of the central wavelength, the increment of temperature will induce a redshift of the central wavelength, which is translated into a decrement of the optical power (or offset DC level after the stage acquisition). Consequently, there is an increment in the sensor’s sensitivity due to the sharper slope of the micro-cavity spectrum for higher wavelengths. Nevertheless, for dynamic applications, as presented here, the temperature variation happens at a much lower rate than the target frequencies, thus, the temperature cross sensitivity can be neglected or attenuated by a bandpass filter. Therewith, in the case of the optical accelerometer, the key information is codified in frequency instead of temporal domain. In the case of the central arterial pulse wave probe sensor, the valuable information is extracted from the shape of the signal which is always normalized and filtered to minimize the temperature cross sensitivity.

## 4. Conclusions

This paper presented a low-cost interrogation technique based on an in-line micro-cavity constructed by recycling optical fiber destroyed by the catastrophic fuse effect. The spectral characteristics of high visibility (23 dB) and a half FSR of 6 nm enabled the FPI micro-cavity to be used as an edge filter. The purposed interrogator was studied in two different sensors with the aim of demonstrating its flexibility in different fields. The resolution of the developed solution was limited by the used ADC module, which was configured with a sample rate of 5000 sps and a measurement range of 5 VDC. In the first case, an optical accelerometer was characterized by comparing its response, measured with the purposed interrogator system, with two different signals: from a reference signal (electronic accelerometer), and a commercial spectrometer with 0.5 pm resolution and sample rate of 945 sps. On the other hand, an optical non-invasive carotid pulse waveform sensor was also tested with the proposed technique, and the result was compared with the same commercial spectrometer as a reference. The achieved resolutions using the optical accelerometer and the optical non-invasive carotid pulse waveform sensor were 3.6 pm and 2.5 pm, respectively. The amplitude and temporal resolution of both sensors’ responses can be considerably improved by increasing the ADC’s sample rate and resolution. Hardware modifications and more detailed analysis of repeatability and resolution will be addressed in future work in order to improve the actual interrogator setup and specifications. In that way, FGBs with lower reflectivity could also be monitored.

## References

[1] Rodríguez C.A., Ribeiro M.R.N., Frizera-Neto A., Castellani C.E.S., Pontes M.J. (2016). Envelope-based technique for liquid level sensors using an in-line fiber Mach-Zehnder interferometer. Appl. Opt..

[2] Alberto N., Tavares C., Domingues M.F., Correia S.F.H., Marques C., Antunes P., Pinto J.L., Ferreira R.A.S., André P.S. (2016). Relative humidity sensing using micro-cavities produced by the catastrophic fuse effect. Opt. Quantum Electron..

[3] Marques C.A.F., Peng G.D., Webb D.J. (2015). Highly sensitive liquid level monitoring system utilizing polymer fiber Bragg gratings. Opt. Express.

[4] Domingues M.F., Tavares C., Leitão C., Neto A., Alberto N., Marques C., Radwan A., Rodriguez J., Postolache O., Rocon E. (2017). Insole optical fiber Bragg grating sensors network for dynamic vertical force monitoring. J. Biomed. Opt..

[5] Antunes P., Dias J., Paixão T., Mesquita E., Varum H., André P. (2015). Liquid level gauge based in plastic optical fiber. Measurement.

[6] Da Silva Marques R., Prado A.R., da Costa Antunes P.F., de Brito André P.S., Ribeiro M.R.N., Frizera-Neto A., Pontes M.J. (2015). Corrosion resistant FBG-based quasi-distributed sensor for crude oil tank dynamic temperature profile monitoring. Sensors.

[7] Culshaw B. (2013). Optical fibre sensors: A current perspective. Open Opt. J..

[8] Lee B.H., Kim Y.H., Park K.S., Eom J.B., Kim M.J., Rho B.S., Choi H.Y. (2012). Interferometric fiber optic sensors. Sensors.

[9] Hill K.O., Meltz G. (1997). Fiber Bragg grating technology fundamentals and overview. J. Lightw. Technol..

[10] Tosi D. (2015). Advanced interrogation of fiber-optic Bragg grating and Fabry–Perot sensors with KLT analysis. Sensors.

[11] Othonos A., Kalli K. (1999). Fiber Bragg Gratings: Fundamentals and Applications in Telecommunications and Sensing.

[12] Holmes C., Carpenter L.G., Gates J.C., Smith P.G.R. (2012). Miniaturization of Bragg-multiplexed membrane transducers. J. Micromech. Microeng..

[13] Holmes C., Gates J.C., Smith P. (2011). Integrated optical differential pressure transducers achieved using thin buckled silica membranes and direct UV written planar Bragg gratings. Sens. Actuators A.

[14] Tiwari U., Thyagarajan K., Shenoy M.R., Jain S.C. (2013). EDF-based edge-filter interrogation scheme for FBG sensors. IEEE Sens. J..

[15] Sengupta D., Kishore P. (2014). Continuous liquid level monitoring sensor system using fiber Bragg grating. Opt. Eng..

[16] Kouroussis G., Kinet D., Mendoza E., Dupuy J., Moeyaert V., Caucheteur C. (2016). Edge-filter technique and dominant frequency analysis for high-speed railway monitoring with fiber Bragg gratings. Smart Mater. Struct..

[17] Zhao Y., Liao Y. (2004). Discrimination methods and demodulation techniques for fiber Bragg grating sensors. Opt. Lasers Eng..

[18] Alfonso J.E., Cárdenas L.G., Triana C.A., Durán M.V. Design of an optical sensing interrogator using an edge filter scheme. Proceedings of the 2013 SBMO/IEEE MTT-S International Microwave & Optoelectronics Conference (IMOC).

[19] Kersey A.D. Interrogation and multiplexing techniques for fiber Bragg grating strain sensors. Proceedings of the Optical Tools for Manufacturing and Advanced Automation.

[20] Cui J., Hu Y., Feng K., Li J., Tan J. (2015). FBG interrogation method with high resolution and response speed based on a reflective-matched FBG scheme. Sensors.

[21] Wu Q., Semenova Y., Sun A., Wang P., Farrell G. (2010). High resolution temperature insensitive interrogation technique for FBG sensors. Opt. Laser Technol..

[22] Roncancio J.S., González N., Cano C.C., Varón M. Low cost optical interrogation system based on FBG sensors. Proceedings of the 2015 SBMO/IEEE MTT-S International Microwave and Optoelectronics Conference (IMOC).

[23] Guo T., Tam H.Y., Albert J. Chirped and tilted fiber Bragg grating edge filter for in-fiber sensor interrogation. Proceedings of the CLEO: 2011-Laser Science to Photonic Applications.

[24] Fallon R., Zhang L., Everall L., Williams J., Bennion I. (1998). All-fibre optical sensing system: Bragg grating sensor interrogated by a long-period grating. Meas. Sci. Technol..

[25] Antunes P.F., Domingues M.F.F., Alberto N.J., André P. (2014). Optical fiber microcavity strain sensors produced by the catastrophic fuse effect. IEEE Photonics Technol. Lett..

[26] André P., Domingues F., Alberto N., Marques C., Antunes P. Recycling optical fibers for sensing. Proceedings of the 2016 SPIE.

[27] Antunes P., Varum H., André P. (2011). Uniaxial fiber Bragg grating accelerometer system with temperature and cross axis insensitivity. Measurement.

[28] Leitão C., Antunes P., Bastos J.M., André P., Pinto J.L. In the trail of a fiber Bragg grating sensor to assess the central arterial pressure wave profile. Proceedings of the Fifth European Workshop on Optical Fibre Sensors.

[29] Leitão C., Antunes P., André P., Pinto J.L., Bastos J.M. (2015). Central arterial pulse waveform acquisition with a portable pen-like optical fiber sensor. Blood Press. Monit..

[30] Leitão C., Antunes P., Pinto J., Mesquita Bastos J., André P. (2016). Optical fiber sensors for central arterial pressure monitoring. Opt. Quantum Electron..

[31] Ikhlef A., Hedara R., Chikh-Bled M. (2012). Uniform fiber Bragg grating modeling and simulation used matrix transfer method. Int. J. Comput. Sci.

[32] André P.S., Facâo M., Rocha A.M., Antunes P., Martins A. Evaluation of the fuse effect propagation in networks infrastructures with different types of fibers. Proceedings of the 2010 Conference on Optical Fiber Communication (OFC/NFOEC), Collocated National Fiber Optic Engineers.

[33] Rocha A.M., da Costa Antunes P.F., Maria de Fátima F.D., Facao M., de Brito André P.S. (2011). Detection of fiber fuse effect using FBG sensors. IEEE Sens. J..

[34] Antunes P., Lima H.F., Alberto N.J., Rodrigues H., Pinto P.M., de Lemos Pinto J., Nogueira R.N., Varum H., Costa A.G., de Brito André P.S. (2009). Optical fiber accelerometer system for structural dynamic monitoring. IEEE Sens. J..

[35] Leitão C., Antunes P., Bastos M.J., Pinto J.L., André P. Optical fiber sensors in arterial pulse waveform acquisition. Proceedings of the Second International Conference on Applications of Optics and Photonics.

